# Microbial effects of part-stream low-frequency ultrasonic pretreatment on sludge anaerobic digestion as revealed by high-throughput sequencing-based metagenomics and metatranscriptomics

**DOI:** 10.1186/s13068-018-1042-y

**Published:** 2018-02-21

**Authors:** Yu Xia, Chao Yang, Tong Zhang

**Affiliations:** 1 School of Environmental Science and Engineering, Southern University of Science and Technology, No. 1008 Xueyuan Blvd, Nanshan, Shenzhen, China; 2 Environmental Biotechnology Laboratory, The University of Hong Kong, Pokfulam Road, Hong Kong, Hong Kong; 3 Department of Microbiology, College of Life Sciences, Nankai University, Tianjin, 300071 China

**Keywords:** Metagenomics, Metatranscriptomics, RNA-seq, Low-frequency ultrasonic pretreatment, Anaerobic digestion, High-throughput sequencing

## Abstract

**Background:**

Part-stream low-frequency ultrasound (LFUS) was one of the common practices for sludge disintegration in full-scale anaerobic digestion (AD) facilities. However, the effectiveness of part-stream LFUS treatment and its effect on AD microbiome have not been fully elucidated.

**Methods:**

Here we testified the effectiveness of part-stream LFUS pretreatment by treating only a fraction of feed sludge (23% and 33% total solid of the feed sludge) with 20 Hz LFUS for 70 s. State-of-the-art metagenomic and metatranscriptomic analysis was used to investigate the microbial process underpinning the enhanced AD performance by part-stream LFUS pretreatment.

**Results:**

By pretreating 33% total solid of the feed sludge, methane yield was increased by 36.5%, while the volatile solid reduction ratio remained unchanged. RNA-seq of the microbiome at stable stage showed that the continuous dosage of easy-degradable LFUS-pretreated feed sludge had gradually altered the microbial community by selecting *Bacteroidales* hydrolyzer with greater metabolic capability to hydrolyze cellulosic biomass without substrate attachment. Meanwhile, *Thermotogales* with excellent cell mobility for nutrient capturing was highly active within the community. Foremost proportion of the methanogenesis was contributed by the dominant *Methanomicrobiales* via carbon dioxide reduction. More interestingly, a perceivable proportion of the reverse electron flow of the community was input from *Methanoculleus* species other than syntrophic acetate-oxidizing bacteria. In addition, metagenomic binning retrieved several interesting novel metagenomic-assembled genomes (MAGs): MAG-bin6 of *Alistipes shahii* showed exceptional transcriptional activities towards protein degradation and MAG-bin11 of Candidatus *Cloacimonetes* with active cellulolytic GH74 gene detected.

**Conclusions:**

In summary, despite the unchanged sludge digestibility, the applied part-stream LFUS pretreatment strategy was robust in adjusting the microbial pathways towards more effective substrate conversion enabled by free-living hydrolyser and beta-oxidation-capable methanogens.

**Electronic supplementary material:**

The online version of this article (10.1186/s13068-018-1042-y) contains supplementary material, which is available to authorized users.

## Background

Anaerobic digestion (AD) is widely applied for the treatment and bioenergy recovery from waste sludge increasingly produced in biological wastewater treatment plants (WWTPs) worldwide. One of the major engineering problems of AD is the long retention times (15–20 days) resulted from the limited conversion efficiency of rigid and non-biodegradable organic structures in sludge [4, 7]. AD performance could be evidently improved by enhancing the rate-limiting hydrolysis step with pretreatment. Ultrasonic (US) pretreatment was the most commonly applied pretreatment method for waste sludge digestion [4, 7, 11]. The effectiveness of key US-pretreatment parameters had been extensively studied during the last 15–20 years, mostly on lab scale [3, 18, 66], with few pilot- and full-scale tests reported [45, 51]. Low-frequency ultrasound (LFUS, < 40 kHz, usually 20 kHz) was the most effective in sludge disintegration as lower frequencies generated larger bubbles with a higher energy release upon implosion [61]. However, there was a noticeable research gap in studying LFUS pretreatment that the lab-scale experiments often pretreated all of the sludge dosed [3, 18, 66], while most of the full-scale installations used part-stream sonication, which consist of treating only a fraction of the sludge stream (usually around 30% of TS [52, 72]). The main advantage of part-stream sonication is to reduce the costs and enhance final sludge dewaterability [11, 45, 51]. Therefore, to reflect the actual effectiveness of LFUS pretreatment on waste sludge digestion, part-stream treatment should be applied in bench-scale digesters to mimic full-scale digestion.

In contrast to the numerous research efforts on operational optimization, the microbial process underpinning the enhanced AD performance by LFUS pretreatment was poorly studied. The effect of LFUS-treated sludge dosage on the AD microbiome was unknown. Moreover, the functionalities of the major populations of AD community digesting LFUS-pretreated sludge had never been elucidated. The limited information at hand shown, like normal AD community, *Bacteroidetes*, *Proteobacteria,* and *Firmicutes* which were the dominant phyla in digester treating LFUS-treated sludge [36, 67]. The increase in sludge digestibility induced by LFUS treatment showed a positive correlation with the relative abundance and richness of *Clostridiales* [67]; however, the reason behind such community shift cannot be explained without information on the roles of different populations within AD community.

Consequently, to elucidate the effectiveness of LFUS pretreatment from both engineering and microbial perspectives, first, bench-scale digesters with part-stream LFUS pretreatment were operated for 3 months. Combined sludge of thickened primary sludge (TPS) and thickened secondary activated sludge (TSAS) was dosed mimicking the real operational condition of a local sewage digestion installation (Sek Wuhui Sewage Treatment Plant, Hong Kong, SAR China). The effectiveness of different part-stream dosage of LFUS-treated sludge was compared in term of volatile solid reduction (VSR) and methane yield. Next, we utilized state-of-the-art high-throughput sequencing (HTS)-based metagenomics and metatranscriptomics to investigate the microbial process in AD digesting LFUS-treated waste sludge. A combination of gene-centric and genome-centric analysis was carried out to identify the key players in rate-limiting hydrolysis and methanogenesis steps of digestion. The genomic information obtained here will add up contextual knowledge on the microbial effect of LFUS pretreatment and enhance our understanding of the microbial interaction in sludge hydrolysis and methanogenesis.

## Methods

### Digester setup and operational parameters

Both TPS and TSAS were collected from Shek Wuhui Sewage Treatment Plant (SWHSTP). The operation of low-frequency ultrasonic pretreatment pilot-trial has started in Mar 2014 at SWHSTP. In addition, Chemical Enhanced Primary Treatment (CEPT) process was applied in this plant with FeCl_3_ dosage at specific gravity = 1.45 g/cm^3^; concentration = 40% w/w to enhance solid removal and inhibit order emission. The mixture of TPS and TSAS, respectively, of 478 and 522 mg/l was used to inoculate four 1-l lab-scale digesters (working volume of 800 ml). After 48 h steady run of the bioreactors, 100 ml of slurry sample was replaced by the same volume of freshly prepared LFUS-pretreated TSAS sample (feed sludge). Two reactors (M1 and M2) were kept as identical controls with supplementation of regular TSAS sample, while the other two reactors (M3 and M4) were supplemented with feed sludge containing LFUS-pretreated TSAS, respectively, taking 23 and 33% of the TS of feed sludge (equivalent to 6 and 9% of the total TS of the reactor) (Table 1). The loading M3 at 6% of total TS represents the current design condition of SWHSTP. During the digestion operation, the digesters were fed with 100 ml freshly prepared feed sludge every 2 days to achieve SRT of 16 days. All digesters were kept at 35 °C and stirring with magnetic stirrer as slow as possible. The digestion process has been operated for 3 months (85 days) with constant pH monitoring (Additional file 1: Figure S1).

**Table 1:** Total solid composition of the digesters sludge and feed sludge

	Control (M1, M2)	M3	M4
TS composition of the digester			
Fraction of LFUS-pretreated TSAS in the digester sludge	0%	6%	9%
TPS (g/l) of the digester:	478	478	478
TSAS (g/l) without LFUS pretreatment	522	459	430
LFUS-pretreated TSAS (g/l)	0	63	92
TS composition of the feed sludge			
Fraction of LFUS-pretreated TSAS in feed sludge	0%	23%	33%
TSAS (g/l) without LFUS pretreatment	278	215	186
LFUS-pretreated TSAS (g/l)	0	63	92

*TS* total solid, *TSAS* thickened secondary activated sludge, *TPS* thickened primary sludge, *LFUS* low-frequency ultrasound

### Ultrasound pretreatment of feed sludge

Feed sludge was prepared by mixing different volumes of LFUS-pretreated TSAS with regular TSAS to a total volume of 100 ml (Additional file 1: Table S1). TSAS was pretreated with ultrasound with sonicator (MCR ltd., Israel). In this study, the ultrasound (20 Hz) horn power was maintained at ~ 63 W (50% amplitude) and ultrasound treatment time is 70 s. The sonifier with these settings generated an ultrasound power dose (US power × time) to TSAS at 4.5 kWh/m^3^, which is equivalent to that of ultrasound system in the pilot-test conducted in SWHSTP.

### Chemical analysis

The operation performance of the digesters, especially the production of CH_4_ gas, was monitored every 4 days. The sludge samples were taken from the bioreactors every 4 days for the chemical analysis. Additional file 1: Table S1 summarizes the measurements of different operational parameters. Briefly, TS and VS are measured using gravity method [6]. The amount of biogas produced in each reactor was measured using a glass syringe. Biogas of 500 µl was sampled to analyze the contents of carbon dioxide, methane, and nitrogen by a gas chromatograph (Hewlett–Packard 5890II, USA) equipped with a thermal conductivity detector and a 2 m × 2 mm (inside diameter) stainless steel column packed with Porapak N (80–100 mesh) [69].

Volatile solid reduction (VSR) was calculated by the following formula:

$${\text{VSR }}\% = {{\left( {{\text{VS}}_{\text{feed}} - {\text{VS}}_{\text{sample}} } \right)} \mathord{\left/ {\vphantom {{\left( {{\text{VS}}_{\text{feed}} - {\text{VS}}_{\text{sample}} } \right)} {{\text{VS}}_{\text{feed}} }}} \right. \kern-0pt} {{\text{VS}}_{\text{feed}} }}\,{\text{in}}\,\% .$$

Specific biogas/methane yield was calculated using below formula:

$${\text{Sp}}.\,{\text{Gas}}\,{\text{ yield}} = {{V_{\text{gas}} } \mathord{\left/ {\vphantom {{V_{\text{gas}} } {({\text{VS}}_{\text{feed}} *{\text{ VSR}} \% *{\text{volume}})}}} \right. \kern-0pt} {({\text{VS}}_{\text{feed}} *{\text{ VSR}} \% *{\text{volume}})}}\quad{\text{in}}\,{\kern 1pt} {{L{\text{-gas}}} \mathord{\left/ {\vphantom {{L{\text{-gas}}} {(g\,{\text{VS-reduced}})}}} \right. \kern-0pt} {(g\,{\text{VS-reduced}})}}.$$

### Total DNA and RNA extraction and Illumina sequencing

As M4 had shown the highest digestive activity in term of methane yield and VS reduction, to investigate the active members of the digestion community, three sludge samples were collected from M4 during operation at 41, 57, and 77 days. These biological triplicates were immediately stabilized into liquid nitrogen for total RNA and corresponding DNA extraction. DNA was extracted using the FastDNA^®^ SPIN Kit for Soil (MP Biomedicals, CA, USA) following the default protocol. For each sample, two replicates of 2 ml sludge (equivalent to approximately 200 µg pellet) were subject to independent DNA extraction. The extracted DNA was then pooled together to get DNA extract of the sample. Next, DNA extract of the three samples (M4 at 41, 57, and 77 days) was subject to independent library construction and subsequent Illumina sequencing at BGI-Shenzhen (BGI-Shenzhen, China).

The extraction of total RNA was conducted immediately after sampling using the PowerSoil Total RNA Isolation Kit (MO-BIO Laboratories, Inc., CA, USA) following the default protocol. The extracted RNA was dissolved in RNase-free water (Sigma, MO, USA) and subsequently treated to remove genomic DNA using the Amplification Grade DNase I kit (Sigma, MO, USA). 1 µg total RNA sample is treated with Ribo-Zero™ Magnetic Gold Kit (Bacteria) (Epicentre, WI, USA) to deplete rRNA. The rRNA-depleted sample was then used to construct Illumina sequencing library using TruSeq RNA Sample Prep Kit v2 (Illumina, CA, USA) at BGI-Shenzhen (BGI-Shenzhen, Shenzhen, China). RNA extract of the three sludge samples was subject to independent library construction and RNA-seq sequencing.

350 and 180 bp insert library was, respectively, constructed from the DNA- and rRNA-depleted RNA. DNA library was sequenced on the Illumina Hiseq2500 platform with PE150 strategy (producing paired-end reads length of 150 bp), while RNA library was sequenced on Hiseq2000 with PE101 strategy (producing paired-end reads length of 100 bp) at BGI-Shenzhen (BGI-Shenzhen, Shenzhen, China).

### Quality control (QC) of Illumina sequence

Raw reads delivered by Illumina sequencing of DNA- and rRNA-depleted RNA were trimmed for sequencing adaptors and noise bases at the end to obtain reads, respectively, of 150 and 100 bp; Next, reads were filtered to remove (1) reads with ambiguous base; (2) reads with low-quality base (quality lower than 20) taking more than 15% of the read length. The post-QC data sets were submitted to MG-RAST server for data sharing (please see Additional file 1: Table S2 for Accession Numbers).

### Metagenomic assembly and gene annotation

Post-QC DNA data sets were assembled together using CLCbio Genomic Workbench (version 6.0.4, CLCbio, Denmark) with default kmer and mapping setting. Only scaffolds longer than 1 kb were kept for gene calling. Next, open reading frames (ORFs) were predicted using MetaGeneMark v3.26 [76]. ORFs were annotated by searching against the NCBI Refseq protein database with rapsearch [71] at E-value cutoff of 1E−5. The tabular results were parsed by MEGAN5 with lowest common ancestor algorithm [26] to assign taxa and corresponding KEGG pathways. HMMER v3.1b [16] was used to search the ORFs against Hidden Markov Models of protein families’ collection of Pfam 27.0 [17] and TIGRFAM 15.0 [21] database.

Phylogenetic affiliation of each contig was first determined based on the taxonomy classifications of genes on the contig. Briefly, if more than 50% of the genes on the contigs were attributed to the same Kingdom, Phylum or Class level taxonomies, then the consensus taxa at given taxonomy level is assigned to this contig and all the associated genes [27]. These criteria were changed to 40% for Order level, 34% for Family level, and 10% for Genus and Species level [27]. Next, the gene-voting-based taxa were further checked by the phylogenetic assignment of PhyloPythiaS^+^ [19] that the PhyloPythiaS assignment would be used if a confliction was observed between these two methods.

### Quantification of gene transcriptional activity

The expression levels of predicted genes were quantified in term of RPKM as previously defined [43]. RPKM-DNA and RPKM-RNA values were, respectively, calculated based on DNA and RNA data sets using RSEM v1.2.28 [35] with default mapping parameters. Sum of RPKM-RNA value of a population was used to evaluate total transcriptional activity of a given population within the community, while relative abundance of this population was compared in term of RPKM-DNA [70]. The ratio of RPKM-RNA to RPKM-DNA, named as MRPKM, was used as a measurement of the absolute activity of a given population within the community [70].

### rRNA sequence identification and transcriptional activities

rRNA sequences were picked from the metagenome and corresponding metatranscriptome by rRNA_hmm.py with default parameters [25]. The picked 16S rRNA sequences of metagenomic data sets were aligned to reference sequences of Greengenes database [40] to obtain operational taxonomic units (OTUs) using close OTU picking algorithm integrated in QIIME [10]. RDP classifier [65] was used to assign taxa for each OTU with confidence threshold of 80%.

### Metagenomic binning

The assembled contigs were binned into population bins following the differential coverage binging pipeline of mmgenome [31]. Briefly, the biological metagenomic triplicate data sets were used to provide differentiate coverage of contigs for the initial binning. Next, the primary bins were further filtered based on genomic feature of tetranucleotide frequency and phylogenetic assignment of PhyloPythiaS^+^ [19]. Eventually to retrieve genomic fragments tending to show inconsistent coverage to rest of the genome, like the 16S rRNA genes, each bin was refined based on the paired-end relationship of read pairs mapped to the contigs within the bin. Relative abundance of each bin was quantified as the number of reads mapped to a bin in percentage of the total number of assembled reads.

## Results and discussion

### Effectiveness of part-stream LFUS pretreatment on AD performance

**Fig. 1:**
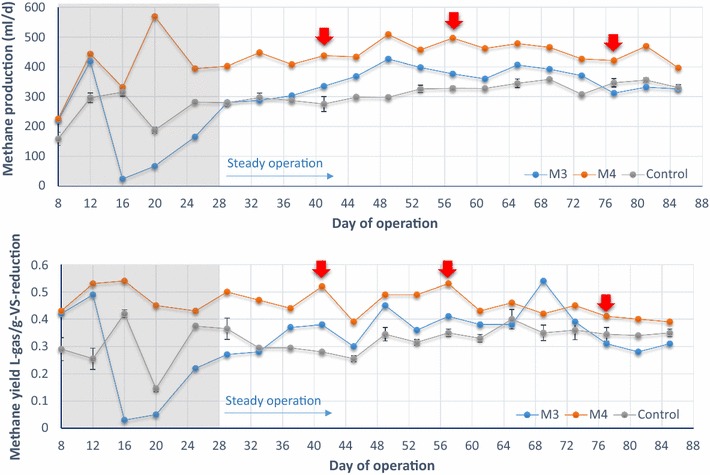
3-month reactor performance in terms of daily methane production (top figure) and methane yield (bottom figure). M3 and M4 group was, respectively, fed with 23 and 33% of LFUS-treated TSAS. Sampling points for metagenomic and metatranscriptomic sequencing were indicated by red arrow

All the digesters reached steady methane generation after 4 weeks (day 25) of inoculation (Fig. 1). Except for bioreactor M3 whose operational pH shifted from 9.4 before day 41 to 7.3; after that, the other reactors all showed stable operational pH around 7.0 (Additional file 1: Figure S2). During the 3-month operation period, the control and ultrasound treatment group (M3 and M4, respectively, fed with 23 and 33% of LFUS-treated TSAS) had significant differences in some key parameters such as daily biogas production and methane (CH_4_) yield (Fig. 1). Complete test results are shown in Additional file 1: Table S3. During steady operation, the biogas production of the two LFUS treatment groups (M3 and M4) was, respectively, 8.7 and 36.5% higher (*p* value < 0.05) than that of the two control reactors (treating regular combined sludge). In the literature, methane yield of digesters treating LFUS-pretreated sludge with full-stream strategy was roughly 40–50% higher than that of the untreated sludge [3, 18, 61, 66]. Therefore, the degree of methane yield increase (36.5% of digester M4 in which 33% of feed sludge was LFUS-pretreated) in our part-stream method was slightly lower than digesters applying a full-stream pretreatment strategy in which all the feed sludge was pretreated. In addition, the average increase on VSR of around 40% by full-stream LFUS pretreatment [3, 18, 24, 46, 61, 72] was not observed in our digesters applying part-stream strategy. In our digesters, the average VSR of the LFUS treatment groups (61.9% for M3 and 60.0% for M4) did not show significant difference from that of control without LFUS-treated TSAS feeding (averagely 59.6%) (Additional file 1: Figure S2). Such unaffected VSR was expectable as only a fraction of feed sludge (33% feed sludge of digester M4) was pretreated, indicating the applied feed loading (LFUS-treated TS taking 9% of the total TS of M4 digester, Table 1) did not decidedly alter the VS digestibility of sludge. However, the evident increased (36.5% of digester M4) methane production with unchanged VSR was noteworthy, suggesting that comparing to full-stream method, part-stream LFUS pretreatment showed more obvious effect on promoting methanogenesis than sludge hydrolysis of AD process. Despite the unaffected sludge solubility, the applied part-stream LFUS pretreatment on feed sludge was enough to induce evident change on chemical composition of the solubilized VS which may facilitate the methanogenesis process and alter the overall microbial pathway within the digester. These observations roused our interests to investigate to what extent the feeding of LFUS-treated sludge could affect the structure of microbial community and what are the active functional populations involves in digestion of LFUS-treated sludge. One more thing to point out was that the sludge used in this study was generated from chemically enhanced primary settling (CEPT process). Such CEPT sludge had shown better digestibility than regular sludge [29]. Consequently, the degree of performance promotion reported here may be higher than other studies apply similar part-stream strategy [12, 52].

### Metagenomic and metatranscriptomic data sets

To reveal the core active populations within the digestion process, three sludge samples were collected from M4 digesters, respectively, at 41, 57, and 77 days of operation. Metagenomic DNA and total RNA were freshly extracted from these biological triplicates for sequencing on Illumina Hiseq platform. Illumina sequencing resulted in totally 9.9 and 10.9 Gb of metagenomic and metatranscriptomic reads (Additional file 1: Table S2). rRNA sequences took 0.2% of the metagenomes constructed. De novo assembly of the metagenomes recovered 401,646 genes (open reading frames) with 42.2% got transcriptional activities detected (Additional file 1: Table S4). Rarefaction analysis based on assembled genes and 16S rRNA sequences of the metagenome data sets both suggested a sufficient coverage of the core populations of the digestive community (Additional file 1: Figure S3).

The metagenome data sets of the three biological triplicates showed high reproducibility in term of RPKM-DNA of the assembled genes (correlation coefficient between replicates > 0.8) (Additional file 1: Figure S4). The highly reproducible gene abundance (in term of RPKM-DNA) in the metagenomic data sets at three sampling times indicated a very stable community structure during steady operation. The transcriptional variation (RPKM-RNA values of genes) among biological duplicates (Pearson’s correlation coefficient of around 0.8) was consistent with the previous evaluation on the reproducibility between metatranscriptomic replicates [62], suggesting reliable metatranscriptome construction in this study. 10,790 genes showed > 4 times variation among metatranscriptomic triplicates. These genes took 6% of the total transcribed genes; among them, genes encoding translation of ribosomal protein showed highest transcriptional variation, suggesting high susceptibility of ribosomal protein synthesis towards environmental change [23, 41].

### Active populations in AD with LFUS pretreatment

**Fig. 2:**
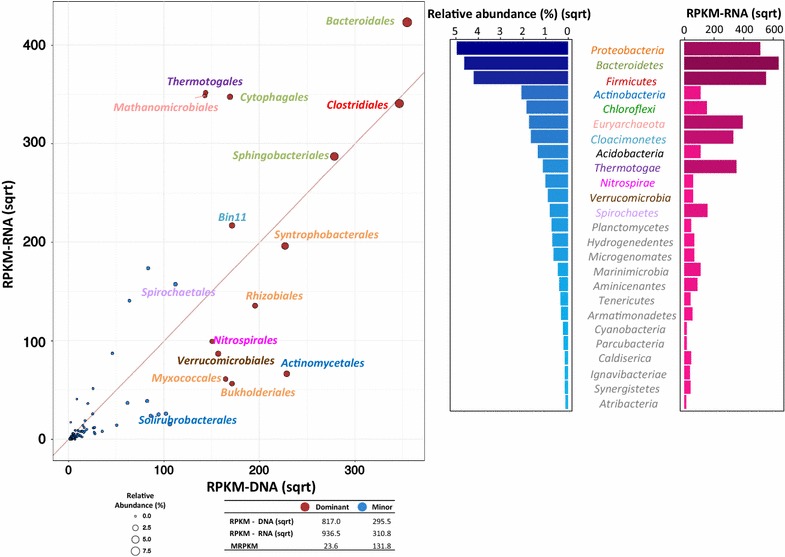
Transcriptional activities of active populations in the CEPT sludge digestion system. The scatter plot on the left shows the active orders within the community. Since active members of *Cloacimonetes* phylum do not have order level taxonomic classification but could be classified at species level (by either PhyloPythiaS^+^ or metagenomic binning analysis), classified species of this phylum are included in the scatter plot. Points of different orders are sized according to their relative abundance within the community; major orders showing > 0.6% abundance are colored in red, while other minor populations are colored in blue. Labels of different orders are colored according to their phylum affiliation as in the right figure. RPKM-DNA, RPKM-RNA, and MRPKM values are respectively summarized for the major and minor populations in the table below scatter plot. The right figure shows the active phyla within the system. Phyla are sorted decreasingly accord to their relative abundance within the community (blue bar), while their transcriptional activities (RPKM-RNA) were shown in the right (pink bar)

Similar to other municipal AD systems [9, 30], *Proteobacteria, Bacteroidetes,* and *Firmicutes,* respectively, taking 24.7, 22.2, and 18.0% of the community were the dominant populations of our AD with LFUS-treated feeding (Fig. 2). Consistent with their prevalence was their active transcriptional activities detected within the community that these dominant phyla contributed 64.5% of the total transcripts. Noteworthy, there were three particularly active populations: *Euryarchaeota*, *Cloacimonetes,* and *Thermotogae* (respectively, taking 3.0, 2.7, and 1.2% of the community). Despite being less prevalence, their transcripts took 25.3% of the whole community transcription, suggesting their important roles within AD fed with LFUS-treated sludge.

The active involvement of *Cloacimonetes*^18^ (a *Candidatus* phylum formerly known as candidatus phylum OP5 and WWE1) was noteworthy. This newly defined population has been widely found in prevalence in AD systems [15, 57, 59]. Genome reconstruction has revealed a putative syntrophic propionate-metabolizing lifestyle of some members of *Cloacimonetes* [44, 48, 57]; however, the available genomic information (one complete genome of *Cloacamonas acidaminovorans* str. Evry [50] and several draft genomes) of this phylum could only covered 1/3 of the *Cloacimonetes* population in our digestion system, suggesting the presence of novel active players within our community. Using multi-dimensional binning strategy, we retrieved the genome (named as MAG-bin11 with 90% completeness and 1.0% contamination) of this novel active member of *Cloacimonetes*. MAG-bin11 was phylogenetically divergent from previously identified lineages of *Cloacimonetes* phylum (Additional file 1: Figure S5). The role of this population will be discussed in detail in subsequent sections.

To gain higher resolution into the roles of different populations, we investigated the major players within the community at Order level based on the trade-off between phylogenetic classification ration and functional interpretability of genes. When interesting expression pattern was discovered in an order, additional effort had been put to retrieve the genomes [more precisely named as the metagenomic-assembled genomes (MAGs)] of the active members within the lineage.

*Methanomicrobiales*, *Bacteroidales*; *Clostridiales*; *Cytophagales,* as well as *Thermotogales* [all showed RPKM-RNA (sqrt) > 300, Additional file 1: Figure S6] were the most active orders identified in the LFUS-treated sludge digestion community. Interesting transcriptional patterns were observed in these active populations. High transcription of genes empowering mobility and chemotaxis was observed in *Thermotogales* (Additional file 1: Figure S6), consistent with our previous finding in the metatranscriptome of thermophilic AD system treating cellulolytic biomass [70]. Such strong transcription was principally (81.8%) contributed by the flagellin protein FlaA which showed 60% amino acid similarity to flagellin protein of *Fervidobacterium changbaicum* and *Fervidobacterium nodosum* [8, 49]. MAG of the active member of *Thermotogales* was retrieved as MAG-bin3. Marker gene-based phylogenetic analysis confirmed MAG-bin3 affiliated with the *Fervidobacterium* genus which contained a variety of hyperthermophilic species that could utilize a wide spectrum of carbohydrate substrates for growth [8, 49]. However, MAG-bin3 showed very low ANI (< 80%) to representative strains of *Fervidobacterium* suggesting its genotype novelty within the genus. The dosage of LFUS-treated sludge might had facilitated its wide spread within our AD community as the increased concentration of soluble substrate by LFUS pretreatment would selectively enrich free-living microbes with higher cellular mobility for effective nutrient capturing. Such metabolic advantage could shed light on the wide spread of this population in AD systems, especially when high content of easy-degradable substrates was available. One thing to point out is that as only roughly 40% of the genes could be assigned to an order-level taxonomy by the annotation methods used (Additional file 1: Table S4), biases caused by the uneven classification ratio of different populations may alter the structure of main active orders revealed in Fig. 2. In addition, there may exist novel active populations that could not be assigned into a given order or could not be recovered by our metagenomic binning protocol due to assembly difficulties.

### Active populations in the hydrolysis pathway

#### Hydrolysis of cellulolytic substrate

**Fig. 3:**
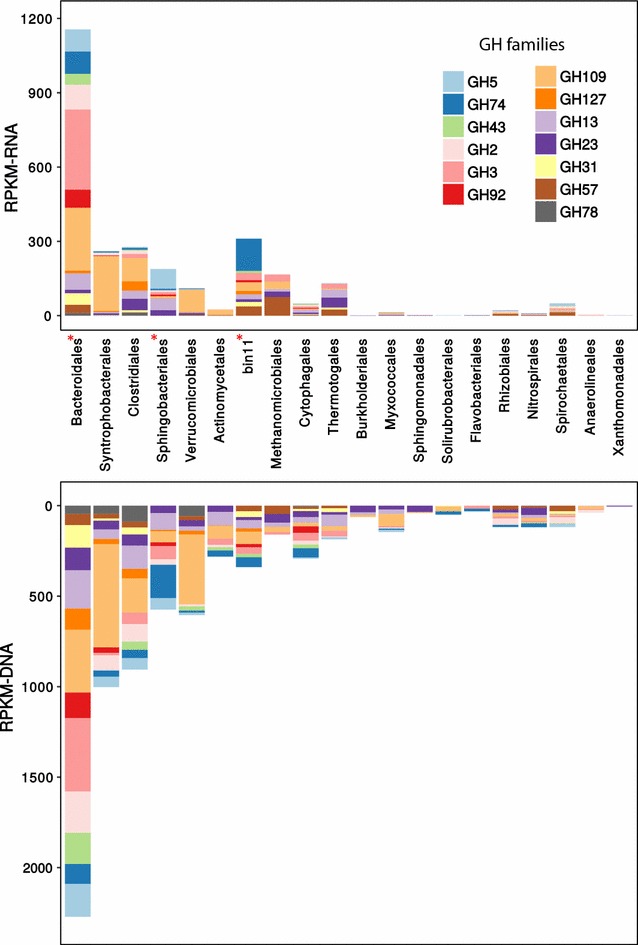
Transcriptional activities (top figure) and relative abundance (bottom figure) of various glycoside hydrolase (GH) families attributed by the major orders of the LFUS-treated sludge digestion community

Hydrolysis of complex polysaccharides, especially the recalcitrant cellulosic component, was regarded as the rate-limiting step for AD process [32]; consequently, the cellulolytic activities of different populations within the LFUS-treated sludge digestion community were studied by comparing the transcriptional activities (in terms of RPKM-RNA) of glycoside hydrolases families (GH families defined by CAZy database). By contributing 24.9% of all the active transcriptions of GH families within the community, *Bacteroidales* played the most active role in carbohydrate hydrolysis within the community. As shown in Fig. 3, active expression of a complete set of hydrolases associated with hydrolysis of cellulosic substrate (including: endoglucanase of GH5 and GH74, hemicellulose of GH43, as well as beta-glycosidase of GH2, GH3, and GH92) were identified in this population. Noteworthy, genes enabling cellulose degradation were more active in *Bacteroidales* than *Clostridiales* in our community (Fig. 3). *Bacteroidales* and *Clostridiales* are both well-known cellulose degraders [37, 38]. However, *Bacteroidales* and *Clostridiales* hydrolyze cellulose with different mechanisms: *Clostridiales* hydrolyze cellulose using cellulosomes [37, 42]. However, *Bacteroidales* do not produce cellulosome; instead, their cellulose hydrolysis is associated with the production of very versatile polysaccharide utilization locis (PULs) [38, 39, 60]. Both cellulosome and PULs are attachment-based cellulose hydrolysis mechanism. However, in our digestion community, only one cellulolytic PULs (PULs that had cellulase-encoding genes located next to or in close proximity to a SusC and SusD gene pair) were identified in an undefined *Bacteroidetes* population with limited level of expression detected (sum of RPKM-RNA of surrounding genes of 36.3). In addition, we observed very low activities of the key building blocks of cellulosomes in *Clostridiales* (RPKM-RNA of Dockerin and Cohesin, respectively, of 0.4 and 110.1). Such reluctant PULs and cellulosome activity indicated a general lack of attachment initiated cellulose conversion in the LFUS-treated sludge digestion system, which was in sharp contradiction to systems digesting rigid cellulosic substrate like raw sludge, grass, or microcrystalline cellulose [20, 70]. These results suggested that despite the physically unchanged digestibility of the treated biomass (as revealed by unaffected VSR% in Additional file 1: Figure S2), the continuous dosage of LFUS-treated sludge (at 33% TS of feed sludge) has gradually altered the hydrolysis pathway by selecting microbes more capable of hydrolyzing cellulosic substrate without attachment.

#### Protein hydrolysis

Since the biodegradation of protein content of the dead cells (residue populations) is associated with the release of ammonia, the major inhibitory compound to both hydrolysis and methanogenesis in AD metabolism, it is indispensable to identify the key protein degraders whose metabolism may cause the ammonia accumulation in the AD systems. As a result, the active populations of protein hydrolysis in the LFUS-treated sludge digestion community were investigated. Protein metabolism (RPKM-RNA of 42708.3) was as active as carbohydrate metabolism (RPKM-RNA of 41789.2) in the community. By comparing the transcriptional activities of key genes associated with SEED subsystem of “Protein degradation”, we observed four populations of *Bacteroidales*, *Clostridiales*, *Methanomicrobiales,* and *Cloacimonas acidaminovorans* actively involved in protein degradation in our digestion community (Additional file 1: Figure S7). As indicated by the different protein degradation-related genes (Additional file 1: Figure S7), polypeptides were broken-down by these populations via miscellaneous metabolic pathways for different motives in the system. Aminopeptidase C (pepC, KO1372, EC 3.4.22.40), taking 55.2% of total transcriptions of protein degradation-related genes, was the most active peptidase within the community. Though the majority (53.3%) of the activities of pepC were contributed by microbes that cannot be phylogenetically assigned, *Alistipes shahii* of *Bacteroidales,* taking 19.3% of pepC activities, was the most active protein degrading species identified in the community. *Alistipes shahii* species is one of the key members of *Bacteroidales* resident in human gut [2, 75]. Its remarkable metabolic capacity towards polypeptides degradation showed here had not been reported elsewhere before, including in isolated strains [58]. Such metabolic advantage in protein degradation might play a vital role in facilitating its widespread in human gut especially in elder adults with more protein-rich diet [33, 74]. A high-quality MAG (named as MAG-bin6 with estimated completeness of 90.7% and contamination of 3.7%) was recovered for *Alistipes shahii*, adding up contextual genomic information to this important species.

Also noteworthy was that genus of *Methanoculleus* and *Candidatus Cloacimonas acidaminovorans* species were another two important protein hydrolyzers within the digestion community. Protein hydrolysis by *Methanoculleus* and *C. acidaminovorans* was, respectively, empowered by the active transcription of AAA-ATPase (PAN) and Proteasome subunit alpha (EC 3.4.25.1). It was the first time that these populations were observed as major protein degraders in AD communities. In addition, energy-dependent proteolysis by Clp protease of an undefined genus of *Peptococcaceae*, taking 37.1% of all the protein degradation activities of *Clostridiales*, was particularly active within our digestion system. Proteolysis by Clp protease was regulatory important for its ability to effectively turnover terminally damaged polypeptides under adverse conditions [13, 22, 34]. The active transcription of Clp protease in *Peptococcaceae* suggested its stressful living condition which was probably imposed by the temporal temperature increase caused by dosage of LFUS-treated feed sludge.

### Active populations in the methanogenesis pathway

**Fig. 4:**
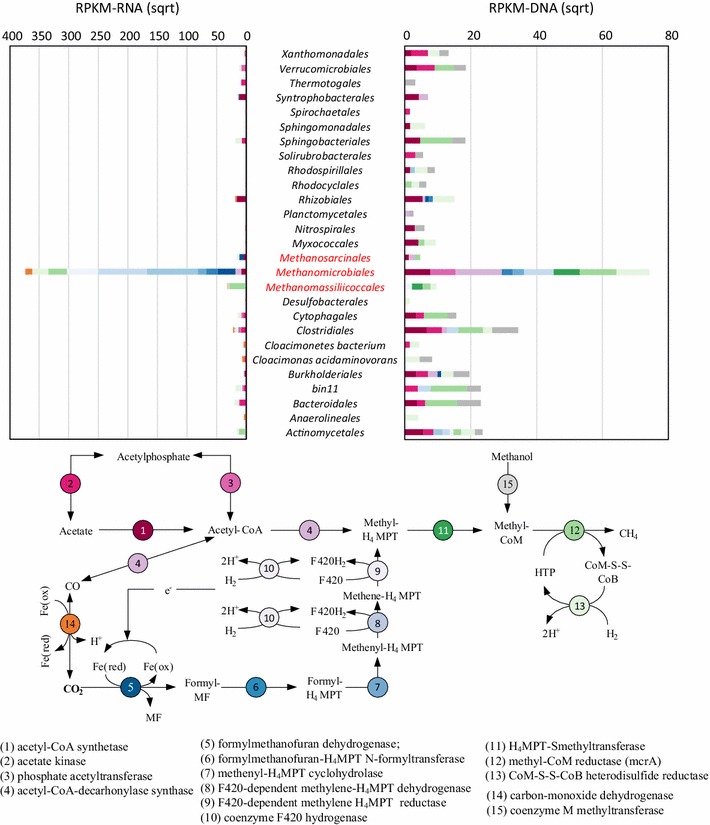
Transcriptional activities (right) and genomic prevalence (left) of genes in the methanogenesis pathway. Genes were classified into various phylogenetic orders, as shown in the top figure, and colored according to their functions in the methanogenesis process (as adopted from the KEGG Methane Metabolism pathway), as shown in the flowchart (bottom figure)

*Methanomicrobiales* and *Methanosarcinales,* respectively, taking 1.04 and 0.14% of the community (Additional file 1: Table S5) were the prevalent methanogenic populations within our LFUS-treated sludge digestion system. Among them, strains of *Methanomicrobiales* were exclusively hydrogenotrophic, reducing CO_2_ into methane with H_2_ as electron donor [47, 63]; while *Methanosarcinales* strains could also produce methane through acetate cleavage [63]. These two archaeal populations often found separately or together as the dominant methanogens in anaerobic digesters [28, 29, 64]. Within our digestion system, the dominant proportion (96.1%) of methanogenic activities of the digestion system was endorsed by *Methanomicrobiales* that the overall transcriptional activity of *Methanomicrobiales* was 14.7-fold higher than that of *Methanosarcinales* (Fig. 4). Correspondingly, we observed the hydrogenotrophic pathway was 10.8-fold more active than aceticlastic pathway in the community (Fig. 4), indicating methane produced in the digestion system was mainly via hydrogenotrophic pathway.

Syntrophic associations between fermentative bacteria and methanogens were important for process stability of hydrogenotrophic methanogenic systems [54, 56, 67]. In anaerobic digesters, syntrophic bacteria produce hydrogen and formate (CO_2_) from its growth substrate (e.g., acetate, propionate, and butyrate). The hydrogenotrophic methanogens consume these products, keeping them at concentration low enough for the overall degradative reaction is thermodynamically favorable [56]. The syntrophic beta-oxidizing population of LFUS-treated sludge digestion system was inferred by the phylogenetic affiliation of the key genes (carbon-monoxide dehydrogenase, EC1.2.7.4, K00198) [56]. Typical syntrophic acetate-oxidizing bacteria (SAOB) were identified by this method. These SAOB included: *Anaerolinea thermophila* of *Anaerolineale* [55], *Candidatus Cloacimonas acidaminovorans* [50], *Cloacimonetes* bacterium JGI 0000039-G13 [53], and some unknown species of *Peptococcaceae* family of *Clostridiales* order (Fig. 4). It was interesting to notice that the majority (68.9%) of beta-oxidation activities were not attributed by these SAOBs. Instead, *Methanoculleus* genus (consist of two species of *Methanoculleus marisnigri* and *Methanoculleus bourgensis*), taking 44.3% of the methanogenesis activities of *Methanomicrobiales*, were the most active syntrophic oxidizer within the community. In our system, active involvement of *Methanoculleus* species in beta-oxidation was evidenced by the transcription of carbon-monoxide dehydrogenase (RPKM-RNA equal to 122.8). A unique metabolic feature of *Methanoculleus* species is their capability to utilize a variety of secondary alcohols as electron donors to reduce CO_2_ to methane [1, 5, 73]. Meanwhile, strains in closely related genus *Methanospirillum* could oxidize ethanol for CO_2_ reduction [68]. The active transcription of beta-oxidation pathway in *Methanoculleus* indicated an important, at least perceptible, proportion of reverse electron transfer within the community took place within the cells of these beta-oxidization-capable methanogens (BOM) other than between their SAOB partners. In the literature, *Methanoculleus* and SAOB were often got concurrently enriched in AD systems with elevated level of ammonia, volatile fatty acids, or other inhibitory intermediate metabolites [14, 54], implying that BOM shared very similar ecological niches with SAOB in AD systems. The functional redundancy on beta-oxidation by BOM and SAOB could help to ensure the stability of hydrogenotrophic methanogenic performance. Our results also suggested that BOM may contribute more in the reverse electron flow of hydrogenotrophic methanogenesis than previously reported.

In summary, foremost proportion of the methanogenic activities of the LFUS-treated sludge digestion system was contributed by the dominant *Methanomicrobiales* via carbon dioxide reduction. More interestingly, the input from *Methanoculleus* species in beta-oxidation was larger than SAOBs of the community. Since BOM and SAOB were concurrently enriched in a variety of AD systems, such major influence of BOM in beta-oxidation revealed here may necessitate a re-evaluation of the syntrophic beta-oxidation pathway in hydrogenotrophic methanogenesis.

## Conclusion

Using state-of-the-art HTS-based metagenomics and metatranscriptomics, microbial mechanisms underpinning the enhanced bioenergy recovery by part-stream LFUS pretreatment were investigated. Results showed that the continuous dosage of LFUS-treated sludge was robust to enrich an effective hydrolyzer community adept of hydrolyzing recalcitrant substrate without attachment. In addition, the vigorous contribution of beta-oxidizing capable methanogens of *Methanoculleus* may play an important role in the promoted methane productivity by LFUS pretreatment.

## Additional file

**Additional file 1: Table S1.** The parameters measured and the corresponding references of analytical methods. **Table S2.** Statistics of metagenomes and metatranscriptomes used in this study. **Table S3.** Performance anaerobic digesters treating different percentage of ultrasound pretreatment TSAS. **Table S4.** Number of genes recovered from the metagenomic data sets (first column) and got transcriptional activities detected (second column). The corresponding annotation efficiency by different databases were listed in the last two columns. **Table S5.** Number of genes of Orders that could be functionally annotated and their transcriptional activities. Orders are sorted descendingly based on their relative abundance within the community. Only orders taking more than 0.1% of the community are shown in the table. **Table S6.** Transcription of the PULs identified in the LFUS-treated sludge digestion system. **Figure S1.** Setup of the for laboratory-scale digesters used in this study. **Figure S2.** 3-month reactor performance in term of pH variation (top figure), volatile solid reduction (VSR) (bottom figure). Sampling points for metagenomic and metatranscriptomic sequencing were indicated by red arrow. **Figure S3.** Rarefaction analysis based on 16S rRNA sequences (bottom figure) and assembled genes (upper figure) of the metagenome data sets. **Figure S4.** Reproducibility based on RPKM-RNA (top figure) and RPKM-DNA (bottom figure) between biological replicates. Regression line between replicates is shown as blue dashed line, while the diagonal line (no variation between replicates) and boundary for 4 times change between replicates are shown as red dash line. Dots are colored according to their RPKM values in corresponding data sets. And the Spearman correlation coefficient R^2^is shown on each subfigure. **Figure S5.** Phylogenetic tree of the available genomes (including metagenome-assembled genomes and complete genome) within *Cloacimonetes* phylum. Maximum-likelihood tree was built based on concatenated alignment of four essential single-copy genes (ESCGs) conserved in single-copy manner among 11 metagenome-assembled genomes (including our bin11) and one finished genome of *Cloacamonas acidaminovorans* Evry. Default protein model of PhyML3.1 was used to construct the tree with 100 bootstraps based on MUSCLE alignment. Boot strap values greater than 50% are indicated at branch points. **Figure S6.** Functions of major orders within the CEPT community(the most prevalent sixteen orders, showing relative abundance > 0.6%). Stacked bar chart shows the transcriptional activities of genes whose functions could be assigned to SEED level 1 functional categories (primary y axis on the left).Only the top 10 most active SEED 1 functions were shown in the figure. The blue line showed the relative abundance of these orders based on RPKM-DNA (secondary y axis on the right). These Orders are sorted descendingly according to their relative abundance. The overall transcriptional activities in term of RPKM-RNA (secondary y axis on the left) were indicated as red diamond. **Figure S7.** Transcriptional activities (top figure) and relative abundance (bottom figure) of key genes involved in the SEED subsystem of “Protein degradation” by different major orders of the LFUS-treated sludge digestion community.

## Abbreviations

LFUS: low-frequency ultrasound/ultrasonic
AD: anaerobic digestion
TS: total solid
VSR: volatile solid reduction ratio
SAOB: syntrophic acetate-oxidizing bacteria
MAGs: metagenomic assembled genomes
WWTPs: wastewater treatment plants
SWHSTP: Shek Wuhui Sewage Treatment Plant
US: ultrasound/ultrasonic
TPS: thickened primary sludge
TSAS: thickened secondary activated sludge
HTS: high-throughput sequencing
CEPT: chemical enhanced primary treatment
QC: quality control
RPKM: reads per kilobase million
GH: glycoside hydrolase
PULs: polysaccharide utilization locis
AcOM: alcohol/acetate-oxidization-capable methanogens
