# Supplementary material for: Microbial effects of part-stream low-frequency ultrasonic pretreatment on sludge anaerobic digestion as revealed by high-throughput sequencing-based metagenomics and metatranscriptomics
Source: Biotechnol Biofuels. 2018 Feb 21;11:47. doi: 10.1186/s13068-018-1042-y (PMC5820786; doi:10.1186/s13068-018-1042-y)
Supplement: Supplementary file 1 — Additional file 1: Table S1. The parameters measured and the corresponding references of analytical methods. Table S2. Statistics of metagenomes and metatranscriptomes used in this study. Table S3. Performance anaerobic digesters treating different percentage of ultrasound pretreatment TSAS. Table S4. Number of genes recovered from the metagenomic data sets (first column) and got transcriptional activities detected (second column). The corresponding annotation efficiency by different databases were listed in the last two columns. Table S5. Number of genes of Orders that could be functionally annotated and their transcriptional activities. Orders are sorted descendingly based on their relative abundance within the community. Only orders taking more than 0.1% of the community are shown in the table. Table S6. Transcription of the PULs identified in the LFUS-treated sludge digestion system. Figure S1. Setup of the for laboratory-scale digesters used in this study. Figure S2. 3-month reactor performance in term of pH variation (top figure), volatile solid reduction (VSR) (bottom figure). Sampling points for metagenomic and metatranscriptomic sequencing were indicated by red arrow. Figure S3. Rarefaction analysis based on 16S rRNA sequences (bottom figure) and assembled genes (upper figure) of the metagenome data sets. Figure S4. Reproducibility based on RPKM-RNA (top figure) and RPKM-DNA (bottom figure) between biological replicates. Regression line between replicates is shown as blue dashed line, while the diagonal line (no variation between replicates) and boundary for 4 times change between replicates are shown as red dash line. Dots are colored according to their RPKM values in corresponding data sets. And the Spearman correlation coefficient R2is shown on each subfigure. Figure S5. Phylogenetic tree of the available genomes (including metagenome-assembled genomes and complete genome) within Cloacimonetes phylum. Maximum-likelihood tree was built based on concatenat [file 13068_2018_1042_MOESM1_ESM.docx]

# Microbial effects of part-stream low-frequency ultrasonic pretreatment on sludge anaerobic digestion as revealed by high-throughput sequencing based metagenomics and metatranscriptomics

Yu Xia^1,2†^, Chao Yang^2,3†^, Tong Zhang ^1,2*^

^1^: School of Environmental Science and Engineering, Southern University of Science and Technology, No. 1008 Xueyuan Blvd, Nanshan, Shenzhen, China

^2^: Environmental Biotechnology Laboratory, The University of Hong Kong,Pokfulam Road, Pok Fu Lam, Hong Kong

^3^: Department of Microbiology, College of Life Sciences, Nankai University, Tianjin 300071, China.

†: These authors contribute equally to this work

## Additional Information

### The parameters measured and the corresponding references of analytical methods

| Parameter | Method | Reference | Sample Type |
| --- | --- | --- | --- |
| TS (g/L) | Gravimetric method | APHA 2540B | Digested sludge |
| VS (g/L) | Gravimetric method | APHA 2540E | Digested sludge |
| pH | Electrometric method | Standard Method 4500-H^+^ | Digested sludge |
| Biogas composition  (N_2_, CO_2_ and CH_4_), % | GC-TCD | HP5890II, USA | Biogas |
| Biogas volume (L) | Collected by air bag and measured by glass syringe | - | Biogas |

### Statistics of metagenomes and metatranscriptomesused in this study

| Sample | Data size, Gb | Average reads length, bp | Post-QC reads number | rRNA reads number | rRNA reads percentage | MG-RAST accession |
| --- | --- | --- | --- | --- | --- | --- |
| Day41 | 3.1 | 100 | 33,701,874 | 726,289 | 2.2% | 4714949  4714944 |
| Day57 | 3.9 | 100 | 33,591,044 | 697,197 | 2.1% | 4714952  471497 |
| Day77 | 3.9 | 100 | 33,612,504 | 942,389 | 2.8% | 4714950  4714941 |
| Day41meta | 3.3 | 150 | 23,805,222 | 59,079 | 0.2% | 4714951 |
| Day57meta | 3.3 | 150 | 23,717,930 | 58,278 | 0.2% | 4714945  4714946 |
| Day77meta | 3.3 | 150 | 23,735,022 | 58,957 | 0.2% | 4714948  4714942 |

### Performance anaerobic digesters treating different percentage of ultrasound pretreatment TSAS.

| Time  Feed sludge | No. of bioreactors | TS (g/l) | VS (g/l) | VSR (%) | pH | Gas yield  L-gas/(g·VS-  reduction) | Methane yield  L-gas/(g·VS-  reduction) | Volume of biogas production (L) | Biogas composition (%) | | |  |
| --- | --- | --- | --- | --- | --- | --- | --- | --- | --- | --- | --- | --- |
|  |  |  |  |  |  |  |  |  | **N_2_** | **CH_4_** | **CO_2_** | |
| 2015/3/26 | M1 | 25.15 | 18.97 | 42.09 | 6.89 | 0.91 | 0.79 | 2.5 | 1.4 | 87.0 | 11.6 | |
|  | M2 | 12.17 | 8.25 | 74.82 | 6.99 | - | - | NA | 5.7 | 64.9 | 29.4 | |
|  | M3 | 18.61 | 13.46 | 58.91 | NA | 0.78 | 0.5 | 3 | 4.9 | 64.0 | 31.1 | |
|  | M4 | 16.9 | 12.17 | 62.85 | NA | 0.61 | 0.36 | 2.5 | 8.6 | 58.8 | 32.6 | |
| 2015/3/30 | M1 | - | - | - | 6.86 | - | - | 1.6 | - | - | - | |
|  | M2 | - | - | - | 6.84 | - | - | 1.2 | - | - | - | |
|  | M3 | - | - | - | 6.87 | - | - | 1.3 | - | - | - | |
|  | M4 | - | - | - | 6.92 | - | - | 2.7 | - | - | - | |
| 2015/4/3 | M1 | 17.38 | 10.94 | 66.61 | NA | 0.41 | 0.35 | 1.8 | 2.3 | 84.6 | 13.1 | |
|  | M2 | 14.37 | 10.7 | 67.34 | 6.71 | 0.34 | 0.23 | 1.5 | 4.4 | 68.1 | 27.5 | |
|  | M3 | 16.67 | 11.76 | 64.1 | 9.47 | 0.64 | 0.42 | 2.7 | 4.1 | 65.4 | 30.5 | |
|  | M4 | 18.18 | 11.58 | 64.65 | 7.04 | 0.66 | 0.43 | 2.8 | 3.0 | 64.5 | 32.5 | |
| 2015/4/7 | M1 | 19.96 | 14.97 | 54.3 | NA | 0.45 | 0.31 | 1.6 | 2.7 | 68.4 | 28.9 | |
|  | M2 | 9.53 | 6.59 | 79.88 | 6.74 | 0.31 | 0.2 | 2 | 1.6 | 63.7 | 34.7 | |
|  | M3 | 21.13 | 15.64 | 52.26 | 9.4 | 0.79 | 0.49 | 2.7 | 8.8 | 62.0 | 29.2 | |
|  | M4 | 21.66 | 16.09 | 50.89 | 7.02 | 0.81 | 0.53 | 2.7 | 4.2 | 65.7 | 30.1 | |
| 2015/4/12 | M1 | 25.69 | 19.28 | 41.15 | 6.85 | 0.67 | 0.44 | 1.8 | 3.6 | 65.8 | 30.6 | |
|  | M2 | 19.16 | 16.18 | 50.61 | 7.07 | 0.57 | 0.4 | 1.9 | 1.8 | 70.2 | 28.0 | |
|  | M3 | 23.98 | 16.98 | 48.17 | 9.35 | 0.38 | 0.03 | 1.2 | 72.2 | 8.2 | 19.6 | |
|  | M4 | 24.2 | 20.53 | 37.33 | 7.1 | 0.82 | 0.54 | 2 | 2.9 | 66.3 | 30.8 | |
| 2015/4/16 | M1 | 10.13 | 7.71 | 76.47 | 6.84 | 0.24 | 0.16 | 1.2 | 1.7 | 67.1 | 31.2 | |
|  | M2 | 8.8 | 6.72 | 79.49 | 6.9 | 0.21 | 0.13 | 1.1 | 1.8 | 63.2 | 35.0 | |
|  | M3 | 10.68 | 8.12 | 75.21 | 9.66 | 0.12 | 0.05 | 0.6 | 28.0 | 45.1 | 26.9 | |
|  | M4 | 9.79 | 7.47 | 77.2 | 7.02 | 0.51 | 0.45 | 2.6 | 3.5 | 87.5 | 9.0 | |
| 2015/4/20 | M1 | 22.8 | 17.6 | 46.28 | 6.86 | 0.59 | 0.38 | 1.8 | 3.0 | 65.0 | 32.0 | |
|  | M2 | 23.31 | 17.95 | 45.21 | 6.93 | 0.57 | 0.37 | 1.7 | 1.7 | 64.1 | 34.2 | |
|  | M3 | 23.06 | 17.3 | 47.19 | 9.22 | 0.36 | 0.22 | 1.1 | 6.7 | 60.2 | 33.1 | |
|  | M4 | 19.47 | 14.43 | 55.95 | 6.98 | 0.65 | 0.43 | 2.4 | 3.5 | 65.7 | 30.8 | |
| 2015/4/24 | M1 | 24.78 | 19.46 | 40.6 | 6.88 | 0.64 | 0.42 | 1.7 | 2.4 | 65.7 | 31.9 | |
|  | M2 | 18.68 | 14.82 | 54.76 | 6.9 | 0.39 | 0.31 | 1.4 | 2.3 | 80.2 | 17.5 | |
|  | M3 | 16.33 | 12.06 | 63.19 | 9.33 | 0.41 | 0.27 | 1.7 | 2.9 | 66.1 | 31.0 | |
|  | M4 | 20.61 | 16.51 | 49.6 | 6.99 | 0.74 | 0.5 | 2.4 | 2.8 | 67.1 | 30.1 | |
| 2015/4/28 | M1 | 15.21 | 10.54 | 67.83 | 6.81 | 0.43 | 0.29 | 1.9 | 2.7 | 66.9 | 30.4 | |
|  | M2 | 20.04 | 14.54 | 55.62 | 6.92 | 0.44 | 0.3 | 1.6 | 2.2 | 69.3 | 28.5 | |
|  | M3 | 16.51 | 12.51 | 61.81 | 9.06 | 0.44 | 0.28 | 1.8 | 3.2 | 64.0 | 32.8 | |
|  | M4 | 18.41 | 13.5 | 58.79 | 6.8 | 0.65 | 0.47 | 2.5 | 2.4 | 71.6 | 26.0 | |
| 2015/5/2 | M1 | 17.27 | 12.52 | 61.78 | 6.98 | 0.44 | 0.29 | 1.8 | 3.4 | 66.4 | 30.2 | |
|  | M2 | 18.67 | 14.43 | 55.95 | 6.92 | 0.46 | 0.3 | 1.7 | 2.8 | 65.0 | 32.2 | |
|  | M3 | 20.89 | 16.5 | 49.63 | 10.17 | 0.58 | 0.37 | 1.9 | 3.1 | 63.9 | 33.0 | |
|  | M4 | 18.77 | 14.22 | 56.59 | 6.98 | 0.65 | 0.44 | 2.4 | 2.5 | 68.0 | 29.5 | |
| 2015/5/6 | M1 | 15.53 | 11.48 | 64.96 | 7.02 | 0.45 | 0.29 | 1.9 | 3.4 | 65.5 | 31.1 | |
|  | M2 | 19.65 | 15.03 | 54.12 | 7.02 | 0.42 | 0.27 | 1.5 | 3.1 | 64 | 32.9 | |
|  | M3 | 19.97 | 15.22 | 53.54 | 9.12 | 0.57 | 0.38 | 2 | 3 | 67 | 30 | |
|  | M4 | 21 | 15.82 | 51.71 | 7.01 | 0.74 | 0.52 | 2.5 | 2.8 | 70 | 27.2 | |
| 2015/5/10 | M1 | 13.64 | 10.24 | 68.74 | 7.08 | 0.4 | 0.27 | 1.8 | 3 | 67 | 30 | |
|  | M2 | 11.82 | 8.67 | 73.53 | 7.1 | 0.37 | 0.24 | 1.8 | 2.5 | 65.8 | 31.7 | |
|  | M3 | 11.45 | 8.17 | 75.06 | 7.46 | 0.45 | 0.3 | 2.2 | 2 | 67 | 31 | |
|  | M4 | 14.76 | 10.38 | 68.32 | 7.03 | 0.56 | 0.39 | 2.5 | 18.5 | 69.3 | 12.2 | |
| 2015/5/14 | M1 | 20.1 | 14.11 | 56.93 | 7.11 | 0.48 | 0.31 | 1.8 | 3.3 | 64.8 | 31.9 | |
|  | M2 | 21.81 | 16.79 | 48.75 | 6.94 | 0.59 | 0.38 | 1.9 | 2.9 | 63.9 | 33.2 | |
|  | M3 | 18.25 | 13.7 | 58.18 | 6.72 | 0.63 | 0.45 | 2.4 | 2.5 | 71 | 26.5 | |
|  | M4 | 16.51 | 11.8 | 63.98 | 7.03 | 0.62 | 0.49 | 2.6 | 2.8 | 78.3 | 18.9 | |
| 2015/5/18 | M1 | 17.1 | 11.91 | 63.64 | 7.08 | 0.46 | 0.3 | 1.9 | 2.85 | 64.84 | 32.31 | |
|  | M2 | 16.96 | 11.82 | 63.92 | 6.84 | 0.5 | 0.33 | 2.1 | 3.17 | 65.5 | 31.33 | |
|  | M3 | 15.23 | 10.45 | 68.1 | 7.42 | 0.54 | 0.36 | 2.4 | 2.91 | 66.36 | 30.73 | |
|  | M4 | 18.62 | 14.24 | 56.53 | 7.01 | 0.73 | 0.49 | 2.7 | 2.83 | 67.69 | 29.48 | |
| 2015/5/22 | M1 | 20.04 | 15.62 | 52.32 | 7.02 | 0.58 | 0.37 | 2 | 2.97 | 63.55 | 33.48 | |
|  | M2 | 17.55 | 12.27 | 62.55 | 6.98 | 0.51 | 0.33 | 2.1 | 2.9 | 64.43 | 32.67 | |
|  | M3 | 19.14 | 14.42 | 55.98 | 7.28 | 0.63 | 0.41 | 2.3 | 2.96 | 65.51 | 31.24 | |
|  | M4 | 18.82 | 13.96 | 57.39 | 7.02 | 0.74 | 0.53 | 2.8 | 2.53 | 70.98 | 26.49 | |
| 2015/5/26 | M1 | 15.57 | 11.39 | 65.23 | 7.02 | 0.49 | 0.31 | 2.1 | 2.97 | 63.85 | 33.18 | |
|  | M2 | 17.99 | 14.22 | 56.59 | 7.02 | 0.54 | 0.35 | 2 | 3.55 | 63.97 | 32.48 | |
|  | M3 | 18.78 | 13.76 | 58 | 7.28 | 0.58 | 0.38 | 2.2 | 3.05 | 65.47 | 31.48 | |
|  | M4 | 15.52 | 11.23 | 65.72 | 6.98 | 0.6 | 0.43 | 2.6 | 2.72 | 71.06 | 26.22 | |
| 2015/5/30 | M1 | 21.34 | 18.39 | 43.86 | 6.97 | 0.7 | 0.45 | 2 | 3.16 | 64.83 | 32.01 | |
|  | M2 | 15.86 | 11.44 | 65.08 | 6.89 | 0.52 | 0.35 | 2.2 | 3.22 | 66.46 | 30.32 | |
|  | M3 | 16.01 | 11.42 | 65.14 | 7.24 | 0.56 | 0.38 | 2.4 | 3.29 | 67.77 | 28.94 | |
|  | M4 | 15.76 | 11.88 | 63.74 | 6.91 | 0.65 | 0.46 | 2.7 | 2.63 | 70.84 | 26.53 | |
| 2015/6/3 | M1 | 13.13 | 9.55 | 70.85 | 7.21 | 0.47 | 0.31 | 2.2 | 3.28 | 65.4 | 31.32 | |
|  | M2 | 16.34 | 14.58 | 55.49 | 7.02 | 0.58 | 0.39 | 2.1 | 3.3 | 67.95 | 28.75 | |
|  | M3 | 20.78 | 18.1 | 44.75 | 7.28 | 0.82 | 0.54 | 2.4 | 3.11 | 65.39 | 31.5 | |
|  | M4 | 13.47 | 10.62 | 67.58 | 7.02 | 0.61 | 0.42 | 2.7 | 3.08 | 68.98 | 27.94 | |
| 2015/6/7 | M1 | 20.42 | 17.56 | 46.4 | 7.19 | 0.66 | 0.41 | 2 | 3.24 | 62.33 | 34.43 | |
|  | M2 | 17.04 | 13.35 | 59.25 | 6.92 | 0.49 | 0.31 | 1.9 | 3.63 | 63.83 | 32.54 | |
|  | M3 | 17.51 | 13.74 | 58.06 | 7.06 | 0.6 | 0.39 | 2.3 | 3.37 | 64.43 | 32.24 | |
|  | M4 | 17.91 | 13.73 | 58.09 | 7.02 | 0.66 | 0.45 | 2.5 | 2.84 | 68.16 | 29 | |
| 2015/6/11 | M1 | 15.06 | 11.85 | 63.83 | 7.15 | 0.5 | 0.31 | 2.1 | 3.51 | 62.26 | 34.23 | |
|  | M2 | 16.86 | 13.13 | 59.92 | 6.67 | 0.59 | 0.38 | 2.3 | 3.28 | 63.79 | 32.93 | |
|  | M3 | 16.4 | 12.44 | 62.03 | 7.35 | 0.52 | 0.31 | 2.1 | 3.97 | 59.34 | 36.69 | |
|  | M4 | 15.78 | 12.15 | 62.91 | 7.02 | 0.61 | 0.41 | 2.5 | 3.09 | 67.35 | 29.56 | |
| 2015/6/15 | M1 | 16.77 | 12.58 | 61.6 | 7.08 | 0.55 | 0.34 | 2.2 | 3.35 | 62.68 | 33.97 | |
|  | M2 | 13.89 | 11.4 | 65.2 | 6.92 | 0.54 | 0.34 | 2.3 | 3.46 | 63.81 | 32.73 | |
|  | M3 | 12.82 | 8.88 | 72.89 | 7.42 | 0.46 | 0.28 | 2.2 | 3.72 | 60.37 | 35.91 | |
|  | M4 | 12.22 | 8.95 | 72.68 | 7.01 | 0.57 | 0.4 | 2.7 | 2.9 | 69.52 | 27.58 | |
| 2015/6/19 | M1 | 16.96 | 13.3 | 59.4 | 7.12 | 0.54 | 0.33 | 2.1 | 3.21 | 60.3 | 36.49 | |
|  | M2 | 17.14 | 14.15 | 56.81 | 7.02 | 0.62 | 0.37 | 2.3 | 3.46 | 59.88 | 36.66 | |
|  | M3 | 15.86 | 12.05 | 63.22 | 7.51 | 0.53 | 0.31 | 2.2 | 3.73 | 59.25 | 37.02 | |
|  | M4 | 15.99 | 12.72 | 61.17 | 7.01 | 0.62 | 0.39 | 2.5 | 2.95 | 63.49 | 33.56 | |

-: value was not measured during the startup of the digesters.

### Number of genes recovered from the metagenomic datasets (first column) and got transcriptional activities detected (second column). The corresponding annotation efficiency by different databases were listed in the last two columns.

| Annotation methods | Total genes number | Expressed gene number | Percentage of genes got annotated | Percentage of expressed genes got annotated |
| --- | --- | --- | --- | --- |
| total | 401,646 | 169,518 | 100% | 100% |
| COG | 1,353 | 683 | 0.3% | 0.4% |
| KEGG | 159,381 | 80,919 | 39.7% | 47.7% |
| SEED | 88,712 | 46,265 | 22.1% | 27.3% |
| PfamA | 258,865 | 128,754 | 64.5% | 76.0% |
| TIGRFAM | 60,508 | 36,619 | 15.1% | 21.6% |
| dbCAN | 13,845 | 7,677 | 3.4% | 4.5% |
| *Kingdom* | 400,623 | 169,152 | 99.7% | 99.8% |
| *Phylum* | 324,252 | 137,395 | 80.7% | 81.1% |
| *Class* | 214,121 | 84,478 | 53.3% | 49.8% |
| *Order* | 149,776 | 62,962 | **37.3%** | 37.1% |
| *Family* | 75,691 | 36,144 | 18.8% | 21.3% |
| *Genus* | 50,921 | 27,310 | 12.7% | 16.1% |
| *Species* | 33,819 | 19,034 | 8.4% | 11.2% |
| Metagenomic binning | 37,332 | 29,475 | 9.3% | 17.4% |

### Number of genes of Orders that could be functionally annotated and their transcriptional activities. Orders are sorted descendingly based on their relative abundance within the community. Only orders taking more than 0.1% of the community are shown in the table.

| Order | Phylum | RPKM-DNA | RPKM-RNA | MRPKM | Abun^1)^, % | Num.^2)^ of gene | Num. of gene with fun^3)^ | Num.of gene with tran^4)^ | Num of gene with fun&tran |
| --- | --- | --- | --- | --- | --- | --- | --- | --- | --- |
| *Bacteroidales* | *Bacteroidetes* | 125710.9 | 179202.1 | 1.4 | 9.04 | 15611 | 12586 | 11145 | 9628 |
| *Clostridiales* | *Firmicutes* | 119995.4 | 116125.0 | 1.0 | 7.22 | 18577 | 14061 | 11986 | 9929 |
| *Sphingobacteriales* | *Bacteroidetes* | 77502.6 | 82508.6 | 1.1 | 6.39 | 10940 | 8513 | 4818 | 4064 |
| *Syntrophobacterales* | *Proteobacteria* | 51399.1 | 38519.9 | 0.7 | 4.17 | 5916 | 4859 | 4937 | 4281 |
| *Actinomycetales* | *Actinobacteria* | 52199.9 | 4437.1 | 0.1 | 2.76 | 21634 | 14704 | 2558 | 1945 |
| *bin11* | *Cloacimonetes* | 29334.4 | 47137.0 | 1.6 | 2.35 | 1959 | 1557 | 1836 | 1547 |
| *Cytophagales* | *Bacteroidetes* | 28620.9 | 120815.6 | 4.2 | 2.21 | 5878 | 4554 | 3017 | 2557 |
| *Verrucomicrobiales* | *Verrucomicrobia* | 24581.5 | 7568.8 | 0.3 | 2.12 | 2552 | 2046 | 2209 | 1878 |
| *Rhizobiales* | *Proteobacteria* | 38113.3 | 18395.9 | 0.5 | 1.8 | 13464 | 10070 | 3467 | 2990 |
| *Burkholderiales* | *Proteobacteria* | 29339.0 | 3202.5 | 0.1 | 1.6 | 9418 | 7319 | 1733 | 1516 |
| *Myxococcales* | *Proteobacteria* | 27084.2 | 3748.3 | 0.1 | 1.44 | 9070 | 6391 | 1948 | 1568 |
| *Thermotogales* | *Thermotogae* | 20619.3 | 123817.6 | 6.0 | 1.21 | 2678 | 2119 | 2331 | 1976 |
| *Nitrospirales* | *Nitrospirae* | 22598.8 | 9915.5 | 0.4 | 1.2 | 6026 | 4477 | 3144 | 2612 |
| ***Methanomicrobiales*** | *Euryarchaeota* | 20394.9 | 121604.0 | 6.0 | **1.04** | 2514 | 1881 | 2223 | 1770 |
| *Spirochaetales* | *Spirochaetes* | 12527.6 | 24759.4 | 2.0 | 0.66 | 2821 | 2265 | 1839 | 1605 |
| *Solirubrobacterales* | *Actinobacteria* | 11259.3 | 237.3 | 0.0 | 0.65 | 3265 | 2435 | 432 | 362 |
| *Xanthomonadales* | *Proteobacteria* | 10394.4 | 680.0 | 0.1 | 0.53 | 4630 | 3456 | 592 | 483 |
| *Sphingomonadales* | *Proteobacteria* | 8883.0 | 639.2 | 0.1 | 0.44 | 4633 | 3767 | 873 | 776 |
| *Rhodocyclales* | *Proteobacteria* | 7394.8 | 571.6 | 0.1 | 0.4 | 2070 | 1775 | 358 | 329 |
| *Anaerolineales* | *Chloroflexi* | 6766.4 | 1515.3 | 0.2 | 0.39 | 1071 | 897 | 648 | 563 |
| *Candidatus Cloacimonas acidaminovorans* | *Cloacimonetes* | 6929.9 | 30172.8 | 4.4 | 0.39 | 1460 | 1139 | 1332 | 1090 |
| *Flavobacteriales* | *Bacteroidetes* | 3824.3 | 1366.5 | 0.4 | 0.31 | 1059 | 721 | 476 | 334 |
| *Cloacimonetes bacterium JGI 0000039-G13* | *Cloacimonetes* | 4081.4 | 19784.6 | 4.8 | 0.23 | 867 | 631 | 778 | 609 |
| ***Methanosarcinales*** | *Euryarchaeota* | 2114.8 | 7637.8 | 3.6 | **0.14** | 669 | 438 | 521 | 399 |
| *Rhodospirillales* | *Proteobacteria* | 2552.9 | 205.5 | 0.1 | 0.13 | 1425 | 1116 | 252 | 225 |

1. Abun: relative abundance within the community
2. Num.: number
3. Fun: function assigned
4. Tran: transcriptional activities detected

### Transcription of the PULs identified in the LFUS-treated sludge digestion system.

| Contig name ^1)^ | Phylum | Order^2)^ | RPKM-RNA | RPKM-DNA |
| --- | --- | --- | --- | --- |
| 3636 | *Firmicutes* | *Clostridiales* | 1482.7 | 234.0 |
| 1786 | *Cloacimonetes* | *bin11* | 422.7 | 167.1 |
| 5496 | *Thermotogae* | *Thermotogales* | 389.4 | 125.5 |
| 1038 | *Firmicutes* | *Clostridiales* | 129.5 | 84.6 |
| 4090 | *Cloacimonetes* | *Cloacimonetes bacterium JGI 0000039-G13* | 120.5 | 77.7 |
| 1594 | *Verrucomicrobia* | *Verrucomicrobiales* | 32.0 | 79.2 |
| 2012 | *Verrucomicrobia* | *Verrucomicrobiales* | 19.0 | 84.5 |
| 67 | *Euryarchaeota* | *Methanomicrobiales* | 18.3 | 90.3 |
| 1593 | *Bacteroidetes* | *Cytophagales* | 2.3 | 13.5 |
| 347 | *Verrucomicrobia* | *Verrucomicrobiales* | 1.9 | 53.7 |
| 1296 | *Bacteroidetes* | *Flavobacteriales* | 0.5 | 54.0 |

1): name of contig containing the PuLs operon

2): strain name was used for *Cloacimonetes* phylum because no order name available for this phylum.

### Setup of the for laboratory-scale digesters used in this study.


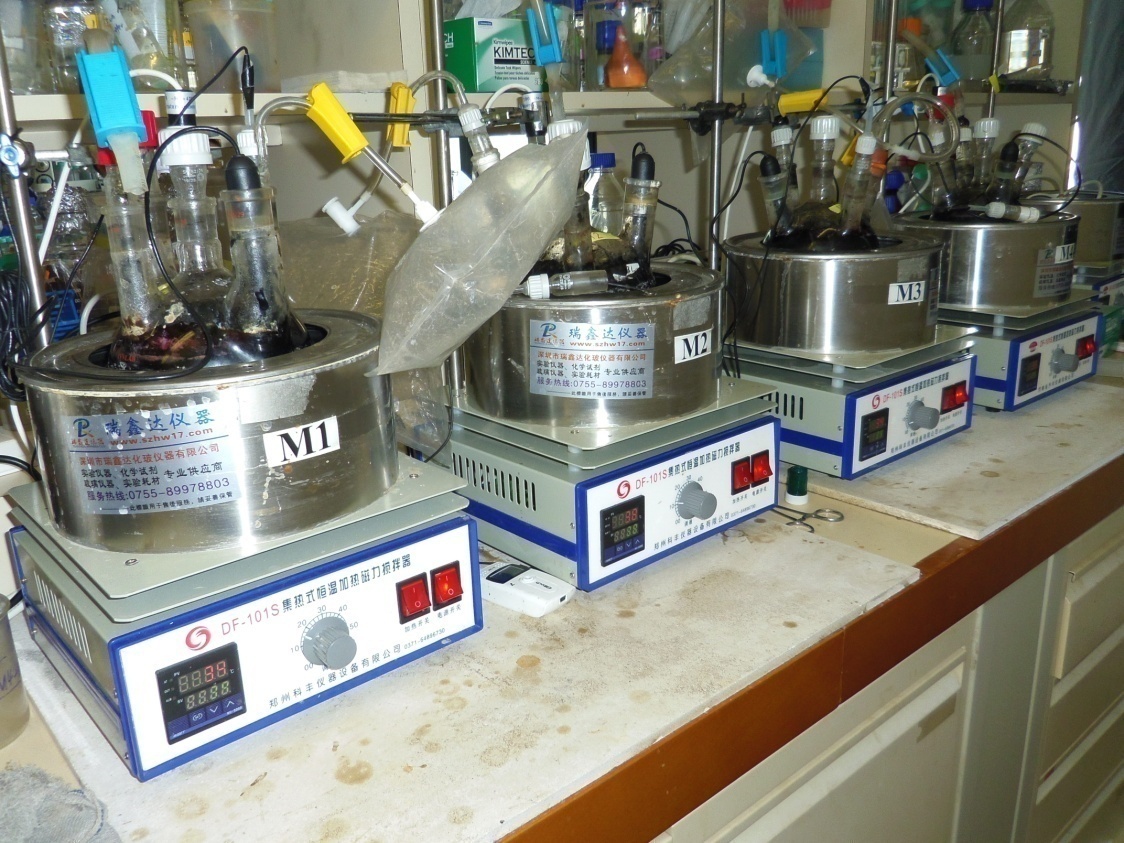


### 3-month reactor performance in term of pH variation (top figure), volatile solid reduction (VSR) (bottom figure). Sampling points for metagenomic and metatranscriptomic sequencing were indicated by red arrow.

### Rarefaction analysis based on 16S rRNA sequences (bottom figure) and assembled genes (upper figure) of the metagenome datasets.

### Reproducibility based on RPKM-RNA (top figure) and RPKM-DNA (bottom figure) between biological replicates. Regression line between replicates is shown as blue dashed line, while the diagonal line (no variation between replicates) and boundary for 4 times change between replicates are shown as red dash line. Dots are colored according to their RPKM values in corresponding datasets. And the Spearman correlation coefficient R^2^is shown on each subfigure.

### Phylogenetic tree of the available genomes (including metagenome-assembled genomes and complete genome) within *Cloacimonetes* phylum. Maximum-likelihood tree was built based on concatenated alignment of four essential single-copy genes (ESCGs) conserved in single-copy manner among 11 metagenome-assembled genomes (including our bin11) and one finished genome of *Cloacamonasacidaminovorans* Evry. Default protein model of PhyML3.1 was used to construct the tree with 100 bootstraps based on MUSCLE alignment. Boot strap values greater than 50% are indicated at branch points.

### Functions of major orders within the CEPT community(the most prevalent sixteen orders, showing relative abundance > 0.6%). Stacked bar chart shows the transcriptional activities of genes whose functions could be assigned to SEED level 1 functional categories (primary y axis on the left).Only the top 10 most active SEED 1 functions were shown in the figure. The blue line showed the relative abundance of these orders based on RPKM-DNA (secondary y axis on the right). These Orders are sorted descendingly according to their relative abundance. The overall transcriptional activities in term of RPKM-RNA (secondary y axis on the left) were indicated as red diamond.

### Transcriptional activities (top figure) and relative abundance (bottom figure) of key genes involved in the SEED subsystem of “Protein degradation” by different major orders of the LFUS-treated sludge digestion community.
